# Exploring the Gene Expression and Plasma Protein Levels of HSP90, HSP60, and GDNF in Multiple Sclerosis Patients and Healthy Controls

**DOI:** 10.3390/cimb46100693

**Published:** 2024-10-19

**Authors:** Igor Sokolowski, Aleksandra Kucharska-Lusina, Elzbieta Miller, Tomasz Poplawski, Ireneusz Majsterek

**Affiliations:** 1 Department of Clinical Chemistry and Biochemistry, Medical University of Lodz, Mazowiecka 5, 92-215 Lodz, Poland; igor.sokolowski@umed.lodz.pl (I.S.); ola_kucharska@wp.pl (A.K.-L.); ireneusz.majsterek@umed.lodz.pl (I.M.); 2 Department of Neurological Rehabilitation, Medical University of Lodz, Milionowa 14, 93-113 Lodz, Poland; elzbieta.dorota.miller@umed.lodz.pl; 3 Department of Microbiology and Pharmaceutical Biochemistry, Medical University of Lodz, Mazowiecka 5, 92-215 Lodz, Poland

**Keywords:** multiple sclerosis, gene expression, ELISA, correlation, GDNF, HSP90, HSP60

## Abstract

Multiple sclerosis (MS) is a chronic neurodegenerative disease characterized by immune-mediated inflammation and neurodegeneration in the central nervous system (CNS). In this study; we aimed to investigate the gene expression and plasma protein levels of three neuroprotective genes—heat shock proteins (HSP90 and HSP60) and glial cell line-derived neurotrophic factor (GDNF)—in MS patients compared to healthy controls. Forty patients with relapsing-remitting MS and 40 healthy volunteers participated in this study. Gene expression was measured using reverse transcription quantitative real-time PCR, and protein levels were assessed via ELISA. The results showed a significant increase in *HSP90* (1.7-fold) and *HSP60* (2-fold) gene expression in MS patients compared to controls, along with corresponding increases in protein levels (1.5-fold for both HSP90 and HSP60). In contrast, *GDNF* gene expression and protein levels were significantly reduced in MS patients, with a 7-fold decrease in gene expression and a 1.6-fold reduction in protein levels. Notably, a non-linear relationship between *GDNF* gene expression and protein concentration was observed in MS patients, suggesting complex regulatory mechanisms influencing GDNF in the disease. The upregulation of HSP90 and HSP60 in MS highlights their roles in immune regulation and stress responses, while the reduction in GDNF indicates impaired neuroprotection. These findings suggest that HSP90, HSP60, and GDNF could serve as biomarkers for disease progression and as potential therapeutic targets in MS, offering promising avenues for future research and treatment development.

## 1. Introduction

Multiple Sclerosis (MS) is a chronic and complex neurodegenerative disease that impacts the central nervous system (CNS). It is generally considered to have an autoimmune origin and ranks among the most prevalent causes of non-traumatic neurological disability in young adults in North America and Europe [1,2,3]. Remarkably, MS is one of the most prevalent chronic inflammatory diseases in adults aged 20–40, with the average age of first diagnosis being approximately 30 years [1]. Notably, MS stands out as one of the most prevalent chronic inflammatory diseases affecting adults aged 20–40, with the average age of diagnosis being around 30 years [1]. Recent reports reveal that the incidence rate of MS is approximately 46,000 in Poland and around 2.8 million globally, and the number of MS patients in Poland is approximately 110 per 100,000 people (https://ezdrowie.gov.pl (URL accessed on 7 August 2024)).

The cause of MS is not fully understood, but it is believed to be the result of a combination of genetic and environmental factors [4]. Studies show that genetic factors play a significant role in the increased occurrence of MS among family members of individuals diagnosed with the disease [5,6]. It is well-documented that a family history of MS is a major risk factor for developing the disease. People with a sibling or parent affected by MS are at a higher risk compared to the general population [7]. The risk of developing MS is higher in first-degree relatives than in second- or third-degree relatives and the general population [7]. Additionally, identical twins have a higher rate of MS concordance than non-identical twins [7]. Pathological changes in the CNS associated with MS include distinct inflammation, the loss of myelin sheaths (demyelination), activation of microglia, and the proliferation of astrocytes leading to gliosis, along with varying degrees of axonal degeneration [8]. The spread of demyelination within the CNS can affect white and gray matter, eventually reducing axonal and neuronal structures [9].

Recent studies have aimed at identifying genes that may play a role in the onset and progression of MS, particularly those with neuroprotective properties. These genes contribute to shielding the nervous system from damage caused by inflammation and other factors related to MS. In this paper, we explore several neuroprotective genes associated with MS, such as heat shock protein 90 (HSP90), heat shock protein 60 (HSP60), and glial cell line-derived neurotrophic factor (GDNF), emphasizing the evidence that supports their potential as targets for MS therapy.

Molecular chaperones are proteins that aid in the folding or unfolding of large proteins or macromolecular complexes, ensuring their proper structure and function. Their specific activities can be categorized as follows: “foldases” assist in properly folding newly synthesized proteins, while “holdases” delay or prevent folding to facilitate processes like protein translocation. These chaperones ensure that proteins fold correctly during or after synthesis and can also help restore structure after partial denaturation, maintaining cellular proteostasis. 

HSP90, a molecular chaperone, plays a crucial role in the maturation of various proteins, including kinases, transcription factors, and E3 ubiquitin ligases. This makes HSP90 a key player in maintaining cellular protein homeostasis. The ATPase cycle of HSP90 undergoes significant structural changes, which are further influenced by cochaperones. These cochaperones regulate client protein interactions, HSP90′s ATPase activity, and its conformational dynamics. Given its pivotal role in cellular regulation, strategies to inhibit HSP90′s function are being explored in diseases like cancer and neurodegenerative disorders [10]. Protein aggregation is a hallmark of several neurodegenerative diseases, including Huntington’s, Alzheimer’s, amyotrophic lateral sclerosis (ALS), and Parkinson’s. In these conditions, misfolded proteins lead to neuronal damage and symptom progression. HSP90 has been implicated in regulating many of the proteins involved in these diseases. However, the exact mechanisms by which HSP90 influences the maturation of these proteins and the therapeutic potential of its inhibition remain under investigation [11,12,13].

The mitochondrial chaperonin HSP60 is a crucial molecule with diverse functions expressed in response to oxidative stress. HSP60 is widely distributed in the brain and associated with various neurological disorders. It has been detected in protein aggregates that are characteristic of neurodegenerative diseases such as Parkinson’s disease (PD), Alzheimer’s disease (AD), and multiple sclerosis, where oxidative stress and mitochondrial dysfunction play vital roles [14]. HSP60 is initially synthesized in the cytosol in response to cellular stress and is subsequently targeted to the mitochondria, where it helps maintain mitochondrial protein homeostasis [15]. It facilitates proper protein folding within mitochondria and assists in refolding denatured polypeptides, an ATP-dependent process involving the cochaperone HSP10 [16]. Recent studies have shown that knocking down HSP60 can lead to reduced mitochondrial activity and increased cell proliferation, highlighting its importance in maintaining mitochondrial function [17].

Neurotrophic factors (NTFs) are proteins essential for neuronal survival, cell proliferation, and differentiation. They are involved in axonal and dendritic growth and regulate synaptic plasticity [18,19,20]. NTFs include neurotrophins like NGF, BDNF, NT-3, NT-4, and GDNF, a part of the GDNF family of ligands [21]. GDNF acts as a neural cytokine, facilitating communication between neurons and their target tissues. It plays a vital role in protecting both the central and peripheral nervous systems from degeneration, as shown in models of neurodegenerative diseases like Parkinson’s disease (PD) and amyotrophic lateral sclerosis (ALS) [19,22]. It is particularly critical in supporting dopaminergic neurons, progressively lost in PD [23]. GDNF also protects noradrenergic and motor neurons, showing potential therapeutic applications for conditions such as Huntington’s disease and ALS [23]. However, the mechanisms by which GDNF exerts these protective effects are still under investigation.

Our previous research examined the relationship between gene expression and protein levels of BDNF, SIRT1, HSP70, HSP27, and NT4 in MS patients and healthy donors [24]. We selected HSP90, HSP60, and GDNF to expand this analysis due to their crucial roles in protein folding and cellular stress responses. HSP70 assists in early-stage folding and prevents aggregation, while HSP90 stabilizes proteins during later stages. HSP90 and HSP70 regulate the heat shock response (HSR), where HSP90 controls HSF-1 activation under stress, leading to HSP70 production to refold damaged proteins [25]. HSP60 works in mitochondria, ensuring proper folding of mitochondrial proteins. This network protects cells from stress-related damage [15]. GDNF, a neurotrophic factor, supports neuronal survival, making it a key marker for studying neurodegeneration [26]. By including these proteins, we aim to provide a more comprehensive understanding of the molecular mechanisms underlying disease progression and cellular stress.

The expression and concentration levels of chaperones and GDNF have been sporadically studied in CNS diseases. This study aimed to assess the potential of HSP90, HSP60, and GDNF as biomarkers for monitoring inflammation and disease progression. We also explored correlations between gene expression and protein levels for these markers through a comparative analysis of MS patients and healthy controls.

## 2. Materials and Methods

### 2.1. Materials

A total of 40 patients with diagnosed relapsing-remitting MS (RRMS), 22 females and 18 males (aged 56 ± 4.5 years), were recruited from the Neurological Rehabilitation Division at the III General Hospital in Lodz. All patients diagnosed with RRMS were in the stable phase of the disease, experiencing remission without any recent attacks. All MS patients included in the study had not received any form of immunomodulatory therapy within the three months following the blood sample collection. The diagnosis of MS in patients was based on the latest McDonald’s criteria (2017 version). A total of 40 healthy individuals without MS, 20 females and 20 males (aged 54 ± 5.5 years), selected for this study, were recruited from the Vadimed Medical Center in Krakow, Poland. The MS patients and healthy donors were matched by age group to ensure comparability (Table 1).

All participants, including both patients and control subjects, enrolled in the study were Caucasians. The study design received approval from the Committee for Bioethics of the Medical University of Lodz in Poland and adhered to the principles outlined in the Declaration of Helsinki. Before the study commenced, informed written consent was obtained from all participants after a thorough explanation of the survey. The clinical diagnosis of multiple sclerosis (MS) in patients was determined based on the McDonald’s criteria (2017 version). Several exclusion criteria were applied, including age below 18 years or above 70 years, severe general health condition of the patient, presence of another neurological disease or autoimmune disease, a personal or first-degree family history of cancer, inability to provide informed consent due to difficulties in logical, verbal communication, presence of severe psychiatric illness hindering informed consent, and active inflammatory acute disease. Before the examination, patients with multiple sclerosis did not exhibit any additional inflammatory diseases or cancer at the time of blood collection.

### 2.2. Gene Expression Analysis

Nine milliliters of blood were drawn by venepuncture into S-Monovette^®^ K3 EDTA, 9 mL, cap red between 8–9 am. According to the manufacturer’s protocol, total RNA was isolated from the PBMC GeneMATRIX Human Blood RNA Purification Kit (EURX Sp. z o.o. Gdansk, Poland) no later than 2 h after sample collection. Total RNA was extracted from 40 patients with MS and 40 subjects without MS. RNA was eluted in 50 μL RNase-free water and stored at −20°C. RNA quality and quantification were measured spectrophotometrically using a Synergy/HT spectrophotometer (Winooski, VT, USA.) and software (Gen5 Version: 2.09.1), applying the 260/280 and 260/230 ratio algorithms. RNA with a 260/280 nm ratio in the range of 1.8–2.0 was considered high quality and was used for further analysis. cDNA was synthesized from 400 ng of total RNA with a High-Capacity cDNA Reverse Transcription Kit (Applied Biosystems™, ThermoFisher Scientific, Waltham, MA, USA) following the manufacturer’s protocol. The cDNA was subjected to a quantitative real-time PCR using the CFX Connect Real-Time System (BioRad, Hercules, CA, USA) with TaqMan PCR Master Mix and TaqMan Gene Expression Assays (Applied Biosystems, Waltham, MA, USA) for *HSP90*. *HSP60* and GAPDH mRNA (*HSP90AA1* Hs00743767_sH, *HSP60* Hs01036753_g1, *GDNF* Hs01931883_s1, and *GAPDH* Hs03929097_g1) were used according to the manufacturer’s instructions. GAPDH expression showed no significant differences between the healthy control and multiple sclerosis groups. Therefore, *GAPDH* was a reliable normalization factor in our analysis, ensuring an accurate comparison of gene expression levels between the two groups. All samples were analyzed in duplicate. In the event of a discrepancy of results (Ct), the result was rejected, and the level of expression of a given gene in a given patient was analyzed again. Ct value is determined by the number of cycles needed to exceed the background signal. The abundance of mRNAs in the studied material was quantified by the 2^−∆Ct^ method.

### 2.3. Evaluation of Protein Levels

Blood was drawn via venepuncture, collecting 9 mL into an S-Monovette^®^ K3 EDTA (Sarstedt, Germany, Nümbrecht) tube with a red cap between 8 and 9 am. Within one hour of collection, the dedicated tubes were processed to separate cells from plasma. This was achieved by centrifuging them in pyrogen/endotoxin-free tubes for 10 min at 1500× *g*, using a refrigerated centrifuge at 4 °C. After plasma collection, samples were aliquoted onto sterile 96-well plates (pyrogen/endotoxin-free) dedicated to each analyzed protein to avoid multiple freeze-thaw cycles. The plasma aliquoted onto the plates was immediately frozen at −20 °C.

The fundamental principle of ELISA involves the specific binding between an antigen (the molecule being detected) and an antibody (a molecule that specifically binds to the antigen). The “enzyme-linked” part of the assay’s name arises from the enzyme attached to the secondary antibody. When provided with its substrate, this enzyme facilitates a color change reaction. The intensity of this color change is proportional to the amount of antigen in the sample, and it can be measured using a spectrophotometer.

The sample characteristics, the target molecule, and the required levels of sensitivity and specificity determine the selection of the ELISA type and specific setup. In this study, Sandwich ELISA kits from Thermo Fisher Scientific were employed for detecting HSP60 and GDNF, while an ELISA kit from Cloud-Clone Corp. was used for HSP90αA1. All assays were performed in strict accordance with the manufacturer’s protocols. Absorbance was measured using the Thermo Scientific™ Multiskan™ FC Microplate Photometer (Thermo Fisher Scientific, Warsaw, Poland), and protein concentration was determined by calculating the average absorbance of each sample based on the standard curve dedicated to each measured protein.

### 2.4. Statistical Analysis

All statistical analyses were conducted with Prism 10 (GraphPad Prism Software (Version 10.2.3), San Diego, CA, USA). The data is presented as the means ± SEM (Standard Error of the Mean) of the conducted experiments, and the distribution of variables was evaluated using the Shapiro–Wilk test. Statistical differences between the data groups were analyzed using the Mann–Whitney U test (for non-normal distribution). Values of *p* < 0.05 were regarded as statistically significant (*p* * ≤ 0.05, *p* ** ≤ 0.01, and *p* **** ≤ 0.0001).

## 3. Results

### 3.1. Gene Expression Levels of Heat Shock Protein HSP (HSP90 and HSP60) and Neurotrophin GDNF in PBMCs in Patients with Multiple Sclerosis

**Figure 1:**
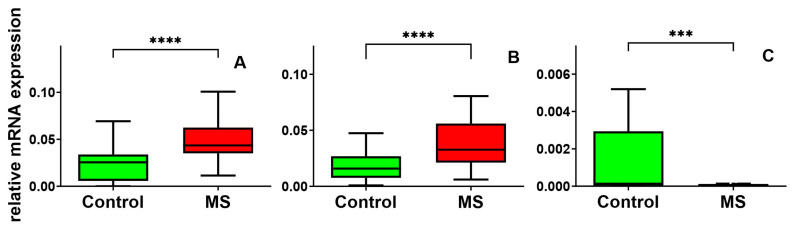
Expression levels of *HSP90* (**A**), *HSP60* (**B**), and *GDNF* (**C**) in PBMCs of MS patients and healthy controls. Statistical analysis of differences between the data groups was performed using the Mann–Whitney U test (**** indicates statistical significance at a *p* < 0.0001) (*** indicate statistical significance *p* < 0.001).

We thoroughly analyzed gene expression levels in peripheral blood mononuclear cells (PBMCs) from patients with MS. We compared them to a control group of healthy adults without known neurological disorders. We focused on evaluating the expression of *HSP90*, *HSP60*, and *GDNF* genes. Our findings revealed significant differences between the two groups. The MS group showed a markedly elevated expression of the *HSP90* gene, with an approximately 1.7-fold increase compared to the control group (median = 0.04350, N = 40 vs. median = 0.02549, N = 40, *p*-value < 0.0001). The 25th percentile in the MS group was 0.03509 compared to 0.005712 in the control group, while the 75th percentile was 0.06235 for MS patients and 0.03387 for healthy controls (Figure 1A). We also observed a 2-fold increase in *HSP60* gene expression in MS patients compared to the control group (median = 0.03274, N = 40 vs. median = 0.01587, N = 40, *p*-value < 0.0001). The 25th percentile in the MS group was 0.02123 compared to 0.007623 in the control group, while the 75th percentile was 0.05599 for MS patients and 0.02695 for healthy controls (Figure 1B). Furthermore, our analysis highlighted significant differences in *GDNF* gene expression between the two groups. MS patients exhibited a decrease in *GDNF* gene expression compared to the control group (median = 1.895 × 10^−5^, N = 40 vs. median = 1.338 × 10^−4^, N = 40, *p* = 0.0007). The 25th percentile in the MS group was 1.680 × 10^−5^ compared to 7.150 × 10^−6^ in the control group, while the 75th percentile was 6.628 × 10^−5^ for MS patients and 2.938 × 10^−3^ for healthy controls (Figure 1C).

### 3.2. Plasma Protein Levels Assessed Through ELISA Testing

**Figure 2:**
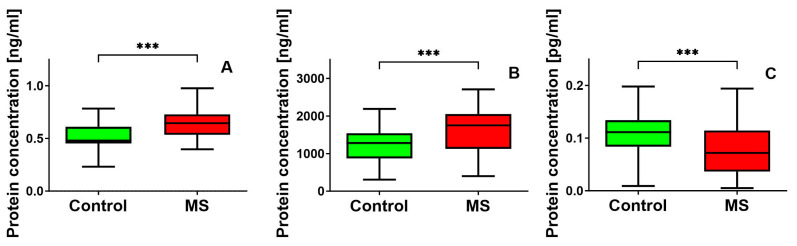
Protein concentrations of HSP90 (**A**), HSP60 (**B**), and GDNF (**C**) in plasma from MS patients and healthy controls. Statistical analysis of differences between the groups of data was performed using the Mann–Whitney U test (*** indicate statistical significance *p* < 0.001).

We also focused on evaluating the concentrations of proteins influenced by previously studied genes. We compared protein levels across two groups. We conducted protein concentration and gene expression analyses on the same participants, providing a unique opportunity to explore the potential relationship between gene expression and protein synthesis directly across these two biological pathways. Firstly, we observed a significant increase in HSP90 levels in MS patients as compared with the control group (median = 0.6455 ng/mL vs. 0.48 ng/mL) as well as HSP60 levels (median = 1752.07 ng/mL, vs. 1280.5 ng/mL). Both increases in HSP90 and HSP60 protein levels in MS patients were statistically significant (*p*-value of 0.0007 and 0.0006, respectively) (Figure 2A). Finally, we examined GDNF protein levels, where a notable difference emerged. MS patients showed lower GDNF concentration than the control group (median = 0.072 pg/mL vs. 0.114 pg/mL). This significant difference was further supported by a *p*-value of 0.0008 (Figure 2C).

### 3.3. Comparison of Gene Expression and Protein Concentrations Between MS Male and Female Patients

Our findings did not reveal significant gene expression or protein concentration differences between MS males and MS females across HSP90, HSP60, and GDNF biomarkers. For *HSP90* gene expression, the MS male group had a slightly higher median value (median = 0.04734) compared to the MS female group (median = 0.04389), but this difference was not statistically significant (*p*-value = 0.6205). The 25th and 75th percentiles for MS males were 0.04244 and 0.07239, respectively, while for MS females they were 0.03887 and 0.06974. Similarly, for *HSP60* gene expression, there was no significant difference (*p*-value = 0.5117), with a median of 0.03857 for MS males and 0.04265 for MS females. The 25th and 75th percentiles were 0.02274 and 0.05719 for MS males and 0.02330 and 0.06288 for MS females. Regarding protein concentration, HSP90 levels showed no significant variation between the groups (*p*-value = 0.5962), with a median of 0.6317 pg/mL for MS males and 0.6593 pg/mL for MS females. The 25th and 75th percentiles were 0.5145 and 0.7697 for MS males and 0.5628 and 0.7214 for MS females. HSP60 protein concentration also showed no significant difference (*p*-value = 0.8148), with a median of 1673.45 pg/mL for MS males and 1752.93 pg/mL for MS females. The 25th and 75th percentiles were 1310.86 and 1973.71 for MS males and 1076.28 and 2103.11 for MS females. Although *GDNF* gene expression and GDNF protein concentrations showed a slight trend towards higher values in MS females, these differences were not statistically significant. For *GDNF* gene expression, the median for MS females was 2.540 × 10^−5^ and for MS males was 1.585 × 10^−5^ (*p*-value = 0.4020). The 25th and 75th percentiles for MS males were 7.375 × 10^−6^ and 2.573 × 10^−5^ while for MS females they were 6.800 × 10^−6^ and 1.167 × 10^−4^. For GDNF protein concentration, while the median for MS males (0.08585 ng/mL) was higher than for MS females (0.05435 ng/mL), this difference did not reach statistical significance (*p*-value = 0.0794). The 25th and 75th percentiles for MS males were 0.04843 and 0.1252, while for MS females they were 0.02188 and 0.07893.

Our results suggest no statistically significant differences in gene expression or protein concentrations between MS males and MS females across the biomarkers examined.

### 3.4. Correlation Between the Gene Expression Level and Protein Concentration

**Figure 3:**
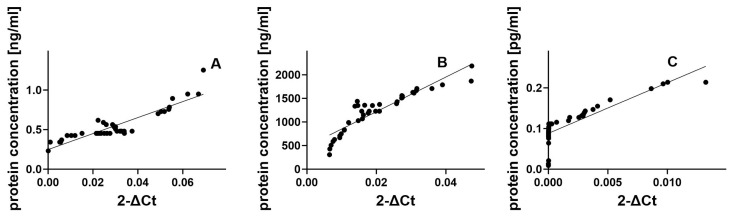
Assessment of correlations between gene expression and protein concentrations of HSP90 (**A**), HSP60 (**B**), GDNF (**C**) in plasma from the healthy donor group. Statistical significance analyzed using Spearman’s rank correlation test (*p* < 0.0001).

Finally, we explore whether the observed changes in gene expression levels of *HSP90*, *HSP60* and *GDNF* in MS patients compared to the control group are connected to parallel alterations in the levels of the proteins expressed by these genes (Figure 3). In the HSP90 control group, we observe a strong and consistent positive correlation (Spearman r = 0.8519) between gene expression and protein concentration (Figure 3A). This indicates that changes in gene expression are closely mirrored by protein-level shifts, though not perfectly synchronized. The highly significant *p*-value (*p* < 0.0001) reinforces the reliability of this association, making it clear that this relationship is far from coincidental. In the HSP60 control group, a notably strong positive correlation (Spearman r = 0.9439) emerges between gene expression and protein concentration (Figure 3B). This tight coupling suggests that protein concentration responds near lockstep as gene expression increases. The exceedingly low *p*-value (*p* < 0.0001) underscores the statistical significance, confirming the robustness of this connection between the two variables. In the GDNF control group, we find an even stronger positive correlation (Spearman r = 0.9669), reflecting a near-perfect alignment between gene expression and protein concentration (Figure 3C). This near-synchronous relationship demonstrates that protein levels are highly dependent on changes in gene expression. With a *p*-value of less than 0.0001, this correlation is highly unlikely to be due to random variation, affirming the strength of the association.

**Figure 4:**
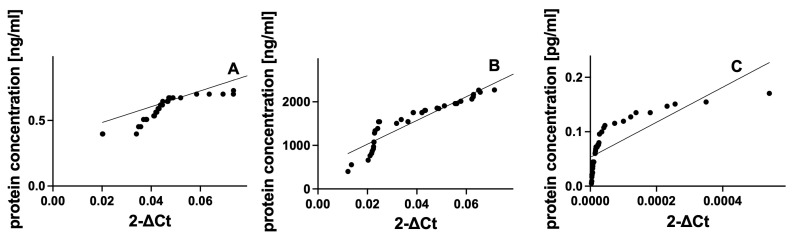
Assessment of correlations between gene expression and protein concentrations of HSP90 (**A**), HSP60 (**B**), GDNF (**C**) in plasma from multiple sclerosis patients. Statistical significance analyzed using Spearman’s rank correlation test (*p* < 0.0001).

Similar to the control group, we observed very high correlations between gene expression and the concentration of the corresponding proteins in the SD group (Figure 4). In the HSP90 MS group, the Spearman coefficient was r = 0.9609 (*p* < 0.0001) (Figure 4A). Higher values of this coefficient were observed for the HSP60 MS group (Spearman r = 0.9978; *p* < 0.0001) (Figure 4B) and the GDNF MS group (Spearman r = 0.9985; *p* < 0.0001) (Figure 4C).

Our analysis revealed significant gene expression and protein concentration differences between patients with MS and the control group. This provides a comprehensive overview of the molecular variations associated with the disease. For *HSP90*, we found a 1.7-fold increase in gene expression in MS patients compared to the control group. This elevated gene expression was accompanied by a 1.5-fold increase in protein concentration, indicating a strong correlation between the two variables. The alignment between the upregulation of *HSP90* gene expression and the corresponding rise in protein levels suggests a clear link between these molecular components.

In the case of *HSP60*, our findings demonstrated a 2-fold increase in gene expression in the MS group compared to healthy individuals. This significant rise in gene activity was closely reflected by a 1.5-fold increase in protein concentration, indicating a direct correlation between higher gene expression and elevated protein levels. The data show that the expression of the *HSP60* gene and its associated protein levels follow a similar pattern, with the changes at the gene level proportionally represented at the protein level.

Conversely, for *GDNF*, we observed a 7-fold decrease in gene expression in MS patients relative to the control group. This substantial reduction in gene expression corresponded with a 1.6-fold decrease in protein concentration, revealing a strong relationship between lower gene activity and reduced protein levels. The decreased protein produced matches the observed reduction in *GDNF* gene expression, further emphasizing the correlation between these two measures.

Our findings across all markers show a consistent correlation between gene expression changes and corresponding protein concentration alterations. The magnitude of these changes, whether increases or decreases, demonstrates a clear relationship between the transcriptional activity at the gene level and the resulting protein concentrations in both the MS and control groups. These observations provide a detailed account of how gene expression levels and protein concentrations vary in parallel across different biomarkers.

## 4. Discussion

Our primary objective was to investigate how HSP90, HSP60, and GDNF protein levels could act as potential markers for evaluating inflammatory processes and disease progression. We explored the possible relationships between alterations in the expression levels of *HSP90*, *HSP60*, and *GDNF* genes and corresponding changes in the protein levels of these markers. This investigation involved a comparative analysis between individuals diagnosed with MS and a control group of healthy volunteers. Understanding the link between these markers’ dysregulated gene expression and protein expression may deepen our insight into MS’s mechanisms, potentially paving the way for new diagnostic methods and therapeutic strategies.

Our research found that HSP90 levels were 1.5-fold higher in MS patients than healthy controls. Evidence from other diseases suggests that HSP90 may play a significant role in the pathogenesis of MS through immune regulation and neuroprotection. Elevated HSP90 levels have been linked to disease severity in systemic lupus erythematosus (SLE) [2,27,28] and idiopathic inflammatory myopathies (IIM) [29]. Similarly, in neurodegenerative diseases like ALS, HSP90 levels are elevated, suggesting a potential protective role in response to ongoing neuroinflammation and neuronal damage [30,31]. The role of HSP90 in AD differs, with decreased levels in AD patients compared to controls [32]. These findings suggest that HSP90 likely acts as a modulator of immune activity and a protective factor against neuronal stress in autoimmune and neurodegenerative conditions, including MS.

Similar to HSP90, we also found that heat shock protein 60 (HSP60) levels were 1.5-fold higher in MS patients than in healthy controls. This evidence suggests that HSP60 may play a role in MS pathogenesis through immune activation and inflammation, similar to its role in other autoimmune and neurodegenerative diseases [14,33,34,35,36,37]. HSP60 could be a marker of inflammation and neuronal stress in MS, and further research is needed to clarify its exact role in the disease. Our previous study showed significant upregulation of other HSPs, HSP70 and HSP27, in MS patients, highlighting their protective roles in response to cellular stress and inflammation [24]. The upregulation of HSP90 and HSP60 in the present study aligns with these earlier findings, suggesting that the broader family of HSPs is consistently involved in modulating stress responses in MS. The increased expression of these chaperones points toward their potential role in mitigating immune-related damage and maintaining cellular homeostasis under inflammatory conditions.

The research indicates that GDNF levels are significantly lower in MS patients compared to healthy controls. This suggests a potential link between GDNF and disease severity in MS, similar to its role in other neurodegenerative conditions like Parkinson’s disease [38] and Alzheimer’s disease [39]. The decreased GDNF levels in MS may indicate impaired neuroprotective support [40], contributing to disease progression.

On the other hand, the downregulation of GDNF in this study mirrors the reductions in neurotrophic factors like BDNF and NT-4 observed previously [24]. This consistent downregulation of neuroprotective factors suggests that MS induces cellular stress and compromises the brain’s ability to repair and protect neurons. The significant reduction in both gene expression and plasma protein levels of GDNF in MS patients further emphasizes impaired neuroprotection, potentially contributing to the neurodegenerative aspect of the disease. We noted the discrepancy between the decrease in *GDNF* gene expression and protein levels. The disparity between the 7-fold reduction in *GDNF* gene expression and the 1.6-fold decrease in protein levels could be partly due to differences in the sensitivity of the assays used. qRT-PCR is extremely sensitive to even small changes in gene expression, especially at low expression levels, which may amplify the fold change observed. In contrast, the ELISA assay for protein quantification may have a lower sensitivity, particularly for low-abundance proteins like GDNF, which could explain the minor fold change in protein levels. Additionally, biological factors such as post-transcriptional regulation and protein degradation may further contribute to this difference. Despite this, the correlation between gene expression and protein levels remains strong, supporting the biological relevance of these findings. We noticed slight differences in the slope and spread of data points, which may suggest variations in protein translation efficiency or regulation between these groups. These differences could reflect altered post-transcriptional regulatory mechanisms in MS patients, where gene expression does not linearly correspond to protein levels. This discrepancy might be due to disease-related factors such as inflammation, cellular stress, or protein degradation pathways that are more active in MS [3]. However, a more detailed exploration of protein synthesis, degradation mechanisms, and post-translational modifications would be required to confirm these potential differences.

Combining the findings from both studies shows that HSPs and neurotrophic factors play crucial yet distinct roles in multiple sclerosis (MS). While HSPs may offer protection against inflammatory damage, the simultaneous decrease in neurotrophic support (e.g., GDNF, BDNF, NT-4) indicates a deficiency in neurorepair mechanisms. These insights suggest that future studies should explore other genes with similar functions, such as SIRT3 genes and a set of anti-inflammatory cytokines (IL-10, IL-4, and IL-37).

Our study has some limitations. Firstly, it had primarily preliminary characteristics, and further research on a larger sample size is necessary to establish potential relationships between changes in the expression levels of *HSP90*, *HSP60*, and *GDNF* genes and MS. A larger sample will also enable the analysis of these relationships in subgroups, taking into account potential confounding factors such as age and others. It will also allow for correlation with the Expanded Disability Status Scale (EDSS) or other variables that quantify disease stage, severity, or cognitive performance. For instance, we deliberately did not gather data on EDSS due to significant variability in how it is rated and the associated functional parameters [41]. This variability can lead to substantial differences in the level of disability. Especially in small sample sizes like ours, this could result in zero counts in some groups and lead to errors in statistical estimation. Furthermore, our analysis is an observational study that only examines gene expression and corresponding protein levels. Therefore, the next step should involve interventional experiments in cell lines or animal models, providing deeper mechanistic insights. We believe this represents a promising direction for future research.

## 5. Conclusions

This study shows significant changes in HSP90, HSP60, and GDNF protein levels in MS patients compared to healthy individuals. In MS patients, HSP90 and HSP60 increase, indicating their involvement in inflammation and immune system regulation. On the other hand, GDNF levels are decreased, suggesting that neuroprotection is impaired in MS. The relationship between *GDNF* gene expression and protein levels in MS patients is not straightforward, highlighting the complexity of GDNF regulation in the disease. These findings suggest that HSP90, HSP60, and GDNF could be used as markers for disease activity and provide insight into potential treatment targets for MS.

## Figures and Tables

**Table 1:** Clinical characteristics of the 40 patients with relapsing-remitting multiple sclerosis (RRMS) and the control group. 10–5.5—no or minor impairment to walking; 6–6.5—requires one or two walking aids; >7—wheelchair mobility or confined to bed.

	MS Group	Control Group
**Number of subjects**	40	40
**Females/males**	22/18	20/20
**Age (years, mean +/− SD)**	56/4.5	54/5.5
**Expanded disability status scale (EDSS) at the stable phase (range)**	5.5 ± 1.0	-

## Data Availability

The data that support the findings of this study are openly available in Mendeley Data at https://doi.org/10.17632/m3mkdz37w6.1 and https://doi.org/10.17632/f4s2sgdz58.1.
